# Differential Modulation of Dorsal Horn Neurons by Various Spinal Cord Stimulation Strategies

**DOI:** 10.3390/biomedicines9050568

**Published:** 2021-05-18

**Authors:** Kwan Yeop Lee, Dongchul Lee, Zachary B. Kagan, Dong Wang, Kerry Bradley

**Affiliations:** Nevro Corp, 1800 Bridge Pkwy, Redwood City, CA 94065, USA; kwan.yeop@nevro.com (K.Y.L.); dlee@nevro.com (D.L.); zack.kagan@nevro.com (Z.B.K.); dong.wang@nevro.com (D.W.)

**Keywords:** spinal cord stimulation, dorsal horn neurons, high frequency, kilohertz, burst

## Abstract

New strategies for spinal cord stimulation (SCS) for chronic pain have emerged in recent years, which may work better via different analgesic mechanisms than traditional low-frequency (e.g., 50 Hz) paresthesia-based SCS. To determine if 10 kHz and burst SCS waveforms might have a similar mechanistic basis, we examined whether these SCS strategies at intensities ostensibly below sensory thresholds would modulate spinal dorsal horn (DH) neuronal function in a neuron type-dependent manner. By using an in vivo electrophysiological approach in rodents, we found that low-intensity 10 kHz SCS, but not burst SCS, selectively activates inhibitory interneurons in the spinal DH. This study suggests that low-intensity 10 kHz SCS may inhibit pain-sensory processing in the spinal DH by activating inhibitory interneurons without activating DC fibers, resulting in paresthesia-free pain relief, whereas burst SCS likely operates via other mechanisms.

## 1. Introduction

Since 1967, spinal cord stimulation (SCS) has been used to manage chronic intractable pain of the trunk and limbs [1]. SCS was derived from the gate control theory: activation of the A-beta fibers in the dorsal columns or dorsal roots would increase innocuous large-fiber input into the spinal pain-gating circuitry [2]. Fundamental to the success of traditional low-frequency (e.g., 50 Hz), SCS is a concept that stimulation-induced paresthesia (defined as any abnormal sensation caused by A-beta fiber activation, including what is often perceived as tingling, buzzing, pins and needles, pressure, etc.) must be experienced by the patient and must overlap with the patient’s painful areas to result in pain relief [3]. The paresthesia overlap with pain was demonstrated to be the single statistically significant technical predictor of therapeutic outcomes by North et al., although perfect coverage with paresthesia did not always ensure a successful outcome [4].

More recently, a new form of ‘paresthesia-free’ SCS (low-intensity 10 kHz SCS) has demonstrated superior long-term pain relief over traditional low-frequency paresthesia-based SCS [5,6]. In contrast to low-frequency SCS, patients receiving clinical 10 kHz SCS do not experience stimulation-induced sensations, and the A-beta-mediated paresthesia–pain overlap from 10 kHz SCS appears uncorrelated with pain relief [6,7]. This suggests that A-beta fibers may not play a significant role in 10 kHz SCS mechanisms, and 10kHz SCS has been termed ‘paresthesia-independent’ [8].

Other SCS strategies have also emerged that attempt to achieve pain relief without generating paresthesia, notably, burst waveforms of different morphologies, e.g., asymmetric biphasically recharged burst and passively recharged burst [9,10]. In a previous study in rodents, we demonstrated that 10 kHz SCS at ostensibly paresthesia-free stimulation amplitudes might preferentially activate inhibitory superficial dorsal horn neurons (SDHN), which are believed to be involved in pain processing [11]. However, the mechanisms for burst SCS are not yet clear [10,12,13,14]. Here, to see if burst stimulation strategies share mechanisms with 10 kHz SCS, we investigated the responses of SDHN, classified as adapting (presumed), excitatory (AE), and non-adapting (presumed) inhibitory (NAI) neurons to different SCS strategies.

## 2. Materials and Methods

### 2.1. Animal Preparation for In Vivo Experiments

**Figure 1:**
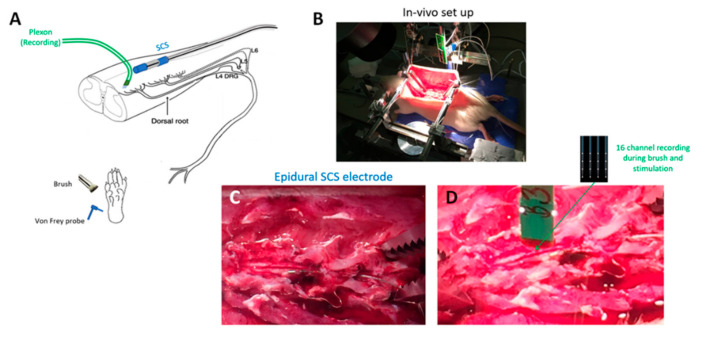
In vivo experimental preparation. (**A**) Schematic showing overall experimental preparation, with sites of spinal stimulation and measurement and afferent mechanical stimuli for dorsal horn neuron characterization. (**B**) Photo of whole animal experimental in vivo setup. (**C**) Photo of dorsal multilevel laminectomy with epidurally positioned spinal cord stimulation mini electrode array. (**D**) Photo of dorsal laminectomy showing multi-electrode Plexon measurement probe prior to insertion into the dorsal cord. SCS electrode also visible.

In vivo experiments (Figure 1) were performed on adult male Sprague Dawley rats obtained from Charles River, Montreal, QC, Canada. Under urethane anesthesia (20% saline; 1.2 g/kg, intraperitoneally), a laminectomy was performed to expose the L4–S1 segment of the spinal cord. The rat was then placed in a stereotaxic frame, and its vertebrae were clamped above and below the recording site to immobilize the spinal cord. The left hind paw was immobilized in plasticine, with the plantar surface facing upwards to receive mechanical stimuli. Rectal temperature was kept at 37 °C using a feedback-controlled heating pad (TR-200; Fine Science Tools, Taunton, MA, USA) throughout the experiment.

### 2.2. Single-Unit Extracellular Recordings

A 4-shank probe with a total of 16 recording microelectrodes (A4 × 4–3mm-50-125-177-A16, NeuroNexus, Ann Arbor, MI, USA) was implanted at the L5 spinal level in the left DH. The array was oriented parallel to the rostrocaudal axis so that each electrode was at roughly the same mediolateral position. The depth of electrode insertion was monitored to estimate electrode position within the presumed Rexed lamina(e).

Electrode shank tips were lowered below the dorsal surface. Since the recording sites were spaced at 50 µm intervals up each shank of the electrode, we estimated the depth of the recorded neurons to be positioned within lamina II–III. Neurons that responded to limb displacement, indicating that they received proprioceptive input, were excluded. Measured extracellular signals of neuronal activity were amplified, and the band-pass was filtered between 500 Hz and 10 kHz, digitized at 40 kHz with the OmniPlex Data Acquisition System (Plexon, Dallas, TX, USA), and stored with stimulus markers on a disk. Single units were isolated using Offline Sorter v3 software (Plexon, Dallas, TX, USA) and analyzed with NeuroExplorer 4 (Plexon, Dallas, TX, USA).

### 2.3. Mechanical Stimulation

Cutaneous receptive fields were identified on the basis of observed spiking evoked by mechanical stimuli applied using a brush or von Frey (VF) filaments to the glabrous skin of the left hind paw. By using weak search stimuli (to avoid peripheral sensitization), we specifically targeted neurons receiving low-threshold afferent input. Each stimulus comprised 10 one-second-long applications of the brush or VF filament (2, 4, 6, 8, and 10 g) repeated at 2 s intervals. Each stimulus was applied twice onto different locations within the receptive field for each test condition.

### 2.4. Spinal Cord Stimulation

**Figure 2:**
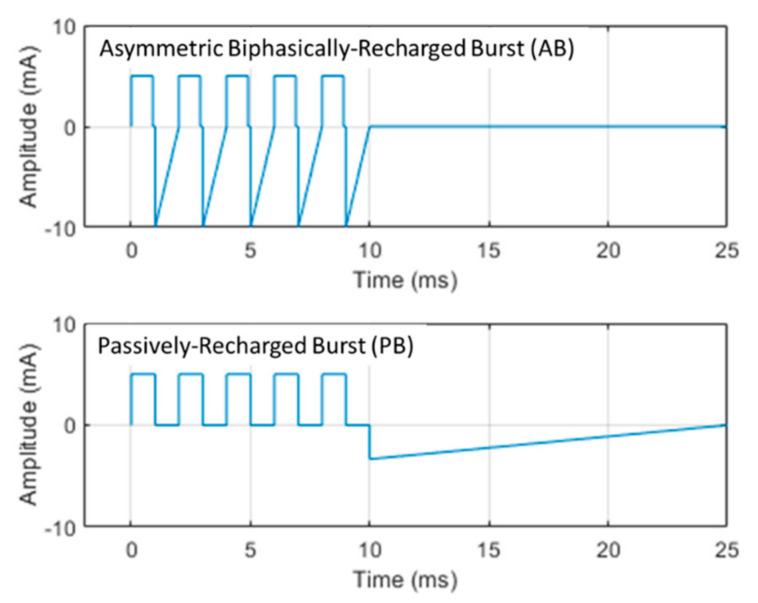
Morphology of different burst waveforms used in experiments. Each burst waveform uses 5 square leading-phase pulses of 1 ms duration separated by 1 ms and delivered at a 40 Hz repetition rate (also known as the intraburst frequency). The name of each burst waveform delineates the form of electrode charge recovery: (**top**) asymmetric biphasically–recharged burst (AB) recovers the electrode charge following each leading-phase pulse using an asymmetric equal-charge recovery waveform; (**bottom**) passively-recharged burst (PB) delays charge recovery until all five leading phase pulses have been delivered. The charge recovery waveform morphology was intended to grossly mimic the classic capacitive-discharge recovery waveform, as known from typical neurostimulators [15]. Example waveforms with amplitudes of 5 mA are shown.

SCS was applied via a miniature in-line multi-electrode array (420 µm diameter, 1 mm-long contact leads interspaced with 1.5 mm-long nonconductive polymers) that was positioned epidurally and ipsilaterally over the L5–L6 dorsal spinal segments (innervating the left hind paw). The SCS electrode was medially positioned within 1–2 mm of the recording microelectrode probe. Two contacts on the miniature SCS lead were used to provide bipolar stimulation. Stimulation was delivered using one of three strategies: 10 kHz, 30 µs symmetric biphasic square pulses, ‘passively–recharged burst’ (PB) waveform, and ‘asymmetric biphasically–recharged burst’ (AB) waveform (Figure 2). All waveforms were current-controlled. 10 kHz stimulation was delivered by a modified version of the same trial stimulator used clinically for 10 kHz SCS therapy (Trial Stimulator, Nevro, Redwood City, CA, USA). Burst waveforms were delivered by a programmable isolated stimulus generator (A-M Systems Model 4100, Sequim, WA, USA). Initially, to determine the motor threshold (MT), a stimulation using each strategy was gradually increased until motor twitch was visible (most often in the hind paw but also observed in the paraspinal musculature/trunk). To allow for clear visualization of muscle activity, the burst stimulation was duty-cycled at 25 ms ON and 2 s OFF, while 10 kHz SCS was duty-cycled at 3 ms ON and 2 s OFF.

### 2.5. General Protocol

Following insertion of the recording microelectrode probe, with 1 h for the electrode to settle in place, mechanical stimuli were applied to the left hind paw to determine which neurons were responsive to innocuous brush stimuli and strong VF (10 g). Next, SCS was applied using the three strategies: 10 kHz, AB, and PB at an intensity of 30% of MT for each strategy, which are reportedly below the sensory threshold, while neuronal responses were recorded [16]. Twenty stimuli of each SCS strategy were administered: each stimulus consisted of a 20 s SCS ON period, followed by a 2 s pause of SCS OFF. SCS was left OFF for at least 5 m between each strategy administration period.

### 2.6. Data Analysis

The SDHN recorded fell into two distinct groups: adapting and non-adapting neurons. Functionally, adapting cells are presumably excitatory and non-adapting cells are inhibitory, based on criteria previously reported [4,17,18,19,20]. Specifically, neurons were identified by their action potential (AP) firing pattern to the 10 g VF filament probing of the hind paw receptive field. We then categorized neurons as either adapting/excitatory (AE) or non-adapting/inhibitory (NAI).

Three types of analyses were used to determine if different SCS strategies demonstrated selectivity to these different neuronal types: firing rate (continuous, across neurons), neuron-type responder (categorical, across neurons), and selective activity ratio (continuous, across animal).

### 2.7. Firing Rate Analysis

From the spiking evoked by each SCS strategy, we calculated the mean firing rate of individual neurons during each stimulus and then subtracted the spontaneous (baseline) firing rate measured from the 10 s period preceding each stimulus. We compared the firing rates in response to the three different strategies for NAI and AE neurons.

### 2.8. Neuron-Type Responder Analysis

With the exception of NAI neurons, most SDHNs do not fire spontaneously in naïve rodents [21]. To establish a meaningful and sensitive responder criterion to SCS, we established a nominal threshold as follows: if the firing rate of the neuron during SCS increased by at least 1 Hz over the preceding baseline firing rate, that neuron was categorized as a responder to the strategy. We then analyzed the differences in responder rates by neuron types and SCS strategy.

### 2.9. Selective Activity Ratio

To determine if a given SCS strategy achieves an aggregate response in a given preparation, we defined and calculated a selective activity ratio: the average firing rate response of all of the NAI neurons found in a given animal was divided by the average firing rate response of all the AE neurons in that same animal. We then compared the selective activity ratio for each SCS strategy across the population of studied animals.

Given that the distribution of the neural responses across neurons and rodents was generally nonnormal (Shapiro–Wilk), neuronal data are reported as medians (quartile range). Comparison of SCS strategies was completed using nonparametric forms of ANOVA, as appropriate to the type of data (continuous: Friedman’s test with post hoc Conover; categorical: Cochran’s Q with post hoc McNemar), with α = 0.05, as corrected using the Benjamini-–Hochberg method.

## 3. Results

**Figure 3:**
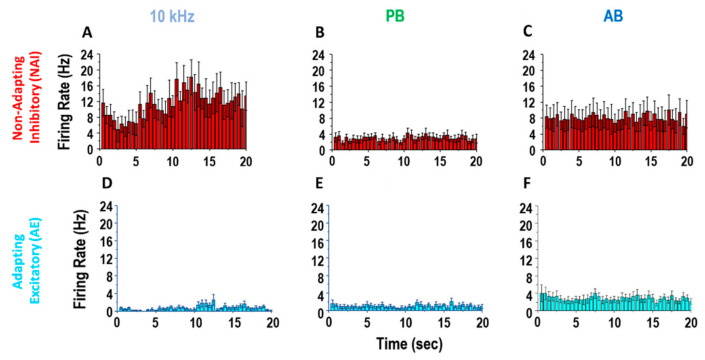
Non-adapting/inhibitory (NAI) and adapting/excitatory (AE) neuron response to SCS strategies. Exemplary firing rate response of typical sub-populations of cells during 20 s SCS: 10 kHz (**A**,**D**), passively-recharged burst (PB) (**B**,**E**), asymmetric biphasically recharged burst) (AB) (**C**,**F**). Top line: non-adapting/inhibitory cells (*n* = 11). Bottom line: adapting/excitatory cells (*n* = 9) (post-stimulus time histogram (bin size: 500ms)).

In 6 rats, we found 21 NAI and 22 AE neurons (3.3 ± 2.1 and 3.5 ± 1.0 per rat, respectively). The 10 kHz SCS produced greater firing responses among NAI neurons compared to PB or AB, whereas it produced minimal firing responses among AE neurons (Figure 3).

### 3.1. Firing Rate Responses

**Figure 4:**
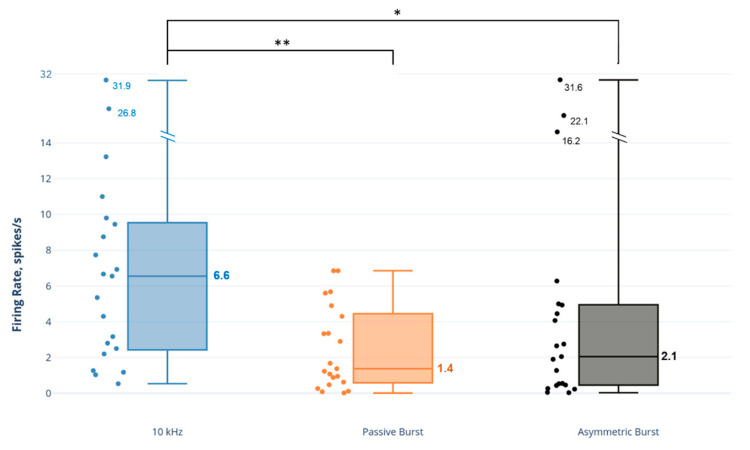
Comparison of dorsal horn non-adapting inhibitory (NAI) neuron firing rates to SCS strategies. Note: axis scale is approximate for firing rate values > 14 spikes/s. * *p* < 0.05; ** *p* < 0.01.

The 10 kHz SCS had a pronounced effect on NAI neurons, approximately 3–4 times greater than the effect of the burst strategies on this neuron type (Figure 4). A comparison of SCS strategies demonstrated that the NAI firing rate response to 10 kHz SCS was statistically significantly and greater than both the PB strategy (*p* = 0.001) and AB strategy (*p* = 0.031).

**Figure 5:**
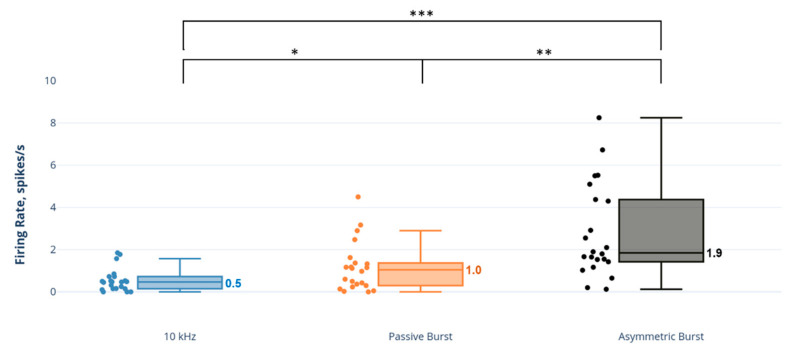
Comparison of dorsal horn adapting excitatory (AE) neuron firing rates to SCS strategies. * *p* < 0.05; ** *p* < 0.01; *** *p* < 0.001.

In contrast, 10 kHz SCS had a minimal effect on AE neurons (Figure 5), with firing rates approximately 2–3.5 times less than the burst strategies and statistically significantly less than the PB strategy (*p* = 0.006) and AB strategy (*p* < 0.001).

### 3.2. Responder Analysis

**Figure 6:**
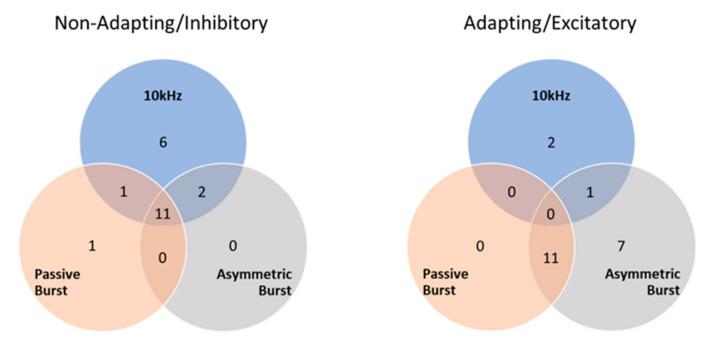
Dorsal horn neuron ‘responders’ to various SCS strategies. Note that one AE neuron did not meet the responder criteria for any SCS strategies.

NAI neurons were activated by all strategies, but most predominantly by 10 kHz SCS (Figure 6) at a statistically significant higher proportion than either burst strategy (10 kHz vs. PB: *p* = 0.03; 10 kHz vs. AB: *p* = 0.01; and PB vs. AB: *p* = 0.80). In contrast, AE neurons were hardly recruited by 10 kHz SCS, while they were more distinctly activated by burst strategies, particularly AB. All strategies recruited AE neurons in statistically significantly different proportions (10 kHz vs. PB: *p* = 0.045; 10 kHz vs. AB: *p* < 0.001; and PB vs. AB: *p* = 0.01). Total responder numbers for NAI and AE neurons for each SCS strategy are shown in Table 1.

### 3.3. Selective Activity Ratio Analysis

**Figure 7:**
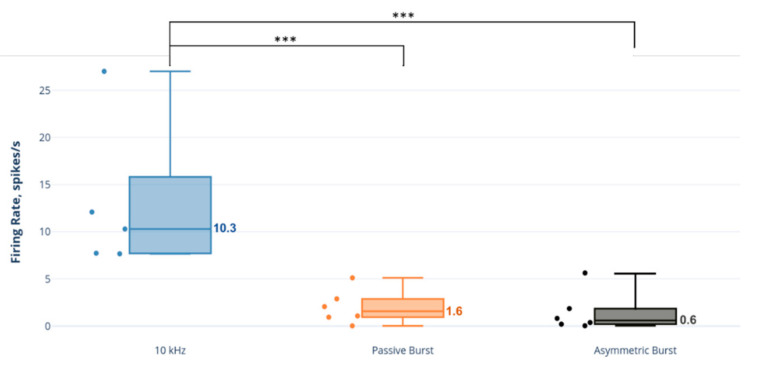
Selective activity ratio: ratio of NAI firing rate to AE neuron firing rate across animals. *** *p* < 0.001.

Based on the selective activity ratio, NAI neurons were statistically significantly more selectively active than AE neurons during 10 kHz SCS relative to burst strategies (10 kHz: 10.3 (7.8–12.1); PB: 1.6 (1.0–2.7); AB: 0.6 (0.2–1.6); comparisons: 10 kHz vs. PB: *p* = 0.001; 10 kHz vs. AB: *p* < 0.001; and PB vs. AB: *p* = 0.29) (Figure 7).

## 4. Discussion

In prior studies, we explored the effects of kHz SCS on DH neurons. Using a very similar in vivo preparation as described here, we compared the responses of DH neurons to 1 kHz, 5 kHz, and 10 kHz SCS frequencies at a variety of stimulation amplitudes. We also used an ex vivo preparation to corroborate the firing rate characterization used in our in vivo preparation, providing evidence that the NAI neurons we studied in vivo were GABAergic. In these studies, we demonstrated that 10 kHz could selectively activate NAI neurons while not significantly activating AE neurons. In addition, we observed that lower kHz settings, such as 1 kHz and 5 kHz, did not effectively activate either type of DH neurons at low stimulation intensities [11]. These results suggested that, when using continuous ‘tonic’ stimulation strategies, very high kHz are necessary to achieve the activation of inhibitory neurons when the stimulation amplitude is below the dorsal column threshold (i.e., ‘paresthesia-free’).

Other stimulation strategies have emerged in the last decade that attempt to provide pain relief without causing significant paresthesia. One of these is the ‘burst’ strategy, where a grouping of stimulation pulses with similar PW and intrapulse intervals (e.g., both 1 ms, yielding an intraburst frequency of 500 Hz), is delivered at intensities near or just below the paresthesia threshold [9,22]. Clinical studies have suggested that many patients (though not all) using burst strategies experience no paresthesia [23,24,25]. Burst strategies have been shown to yield statistically superior pain relief over low-frequency paresthesia-based stimulation [26]. Spinal mechanisms of burst SCS have been explored in vivo; some studies have suggested that burst strategies do not activate GABAergic mechanisms in the spinal cord, while others suggest that GABA is involved in burst SCS [10,27,28,29]. However, these studies did not provide direct evidence of the neural elements involved by the burst strategies.

Clinically, since 10 kHz SCS is a truly paresthesia-independent strategy and burst SCS typically generates no paresthesia, we sought to determine, as some have proposed, whether these strategies may work via similar mechanisms [8,30]. If true, our supposition was that 10 kHz and burst SCS would activate or modulate neurons in the DH in a similar manner.

In general, we found that 10 kHz SCS was highly selective in the activation of NAI neurons in the superficial DH compared to burst strategies. The frequency 10 kHz engendered NAI neuron firing rates 3–4 times greater than the burst modes; 10 kHz drove NAI neurons to a median rate of 6 spikes/s compared to 1–2 spikes/s for the burst strategies. In contrast, AE neurons, while not strongly activated by any strategy (firing rates ≈1–2 spikes/s), were barely activated by 10 kHz, significantly less than both burst waveforms.

We also characterized the differential neuron responses using a responder analysis, where an SCS strategy that elevated the neuron firing rate by ≥1 spike/s indicated that neurons responded to the strategy. Again, we observed that 10 kHz SCS was selective, activating 95% of the found NAI neurons, while only activating 9% of the found AE neurons. Both burst strategies were less discriminating, activating both NAI neurons (about half of the found total) and AE neurons (50–86% of the found total) more equally.

The firing rate and responder analyses both suggested a selective activating effect in which NAI neurons were recruited and driven more strongly compared to AE neurons by 10 kHz SCS, whereas the responses to burst strategies were more equivalent between neuron types. Since these analyses were performed across all found neurons, we sought to determine if this selectivity was true on a per-specimen basis. The selective activity ratio, defined as the firing rate of NAI neurons to the firing rate of AE neurons in a given animal, was conceived as a translational metric. Examining the effect of SCS on DH excitatory and inhibitory neurons separately may be crucial, as simultaneous activation of these neurons might cancel each other’s influence on pain processing by projection neurons and thus produce no apparent aggregate influence. In these studies, we observed that 10 kHz SCS had a far larger selective activity ratio than either burst strategy across animals, approximately 6–17-fold.

We do not know the actual biophysical neural activation mechanisms of 10 kHz or burst SCS, but given that the 30% MT SCS amplitude and pulse width combination for the study were presumably below the activation threshold of nearby large myelinated axons, we focus on the temporal aspects of the different stimulation types. Temporal summation (TS) is a neurophysiologic phenomenon that relates to the cumulative effect of multiple stimuli on neural function [31]. TS can occur in different locations of the neuron; the most commonly studied form is synaptic temporal summation, where multiple action potentials, upon reaching the terminus of a neuron in rapid succession, release a larger amount of neurotransmitter per unit time than if the action potentials arrive at a slower rate. For this form of TS to be effective, the time period between APs arriving at the terminal need only be somewhat shorter than the decay time of the neurotransmitter within the synaptic cleft. In spinal neurons, this is typically at least several milliseconds. This implies that the burst frequency we used of 500 Hz (period = 2 ms) could easily drive TS at synapses that feed the neurons we studied [32,33].

Another form of TS can occur at the membrane itself, where the rapid sequence of extracellular SCS pulses can depolarize the membrane gradually by activating nonlinear mechanisms embedded in the membrane; this can have several effects. For example, if the membrane potential increases to reach the threshold after a number of rapid SCS pulses, it may generate an AP [34]. Additionally, if the sequence of SCS pulses merely increases the depolarization potential in the presynaptic terminal membrane, it can alter the effect of arriving action potentials on neurotransmitter release at the neuron terminal, e.g., by primary afferent depolarization [35]. This form of TS is thus dependent upon the decay time of the membrane potential after being stimulated, which is much shorter than for synapses, perhaps by the order of 300 µs [36]. This suggests that high kHz frequency stimulation pulses would be required to drive this form of TS or ‘membrane summation’.

At the intensities we studied, 10 kHz SCS appears to activate neuronal structures that cause NAI firing. These structures could be the membrane of the NAI neurons themselves or the presynaptic dendrites that synapse onto the NAI neurons, driven to reach the AP threshold. Alternatively, 10 kHz SCS could depolarize, but not fire, inhibitory terminals that feed NAI neurons, resulting in NAI neuron disinhibition and unveiling greater spontaneous activity. However, the drive of presynaptic terminals by SCS, a field-driven effect, might be expected to affect excitatory and inhibitory terminals alike. We observed a high degree of NAI selectivity with 10 kHz SCS, so the minimal activation of AE neurons by 10 kHz SCS makes the drive of presynaptic terminals seem less likely. Further electrophysiological studies are necessary to understand whether 10 kHz SCS has a direct effect on NAI neurons.

In contrast, burst waveforms, which were essentially designed to enhance synaptic throughput, could drive both types of neurons in a non-selective fashion, though not strongly, which generally matches our results [9]. This suggests that burst strategies might work through the activation of axons or axon terminals, which then indirectly drive both interneuron types that we studied. Alternatively, burst waveforms could drive both NAI and EA neurons directly. Cellular-level electrophysiologic work could elucidate these hypotheses.

### Limitations

Stimulation intensity: we explored only a single stimulation intensity, 30% MT, in this study. Our previous work with 10 kHz SCS suggests that a range of 10–30% MT is a reasonable, clinically translatable range, where no dorsal columns would be activated and, thus, no paresthesia would be experienced. For consistency, we also used 30% MT for the burst strategies. When burst is used clinically, the reported range of stimulation has been suggested to be ‘below’ the paresthesia threshold in many, but not all, patients, suggesting that the effective stimulation intensity of burst might be closer to the paresthesia threshold. Song et al. reported that they could detect changes in rodent behaviour with SCS when delivered near 40–50% MT [16]. Thus, our use of 30% MT for the burst waveforms would likely be relevant for patients using burst where paresthesia is not experienced. Other researchers have recently studied burst at 33% MT in preclinical models and observed improvements in paw withdrawal thresholds, albeit less than those observed at higher stimulation intensities, closer to a presumed dorsal column stimulation threshold [37]. We also note that much of the preclinical work in which burst waveforms have been studied has used SCS applied at 90% MT, which most certainly would be expected to activate dorsal columns and, thus, to generate paresthesia [28,29,38].

Stimulation waveshape: our burst waveforms were approximations to those that have been presented in public forums. We made our best efforts to match the key parameters of each waveform to what has been presented, but we cannot guarantee that subtle differences between the stimulation waveforms we used and those used in other experiments would have important effects. Other labs have explored different morphologic parameters representing large changes to the shape of the burst waveform (i.e., doubling the number of burst pulses, the pulse width of leading phase, the pulse frequency, etc.). These studies suggested that large changes to the burst waveshape significantly, but not profoundly (e.g., 0–30% changes in neural firing), affected the measured outcomes they studied [28]. Thus, we feel that any subtle waveform differences in our applied burst waveforms compared to those studied clinically would only have minor influences on the possible translatability of our conclusions.

Short-term study: our experiments were acute electrophysiologic studies of ‘immediate’ neuron response to the applied waveforms. These results may not be predictive of longer-term outcomes, as might be revealed in behavioral studies or electrophysiologic studies performed after many hours or days of applying these strategies. Other labs have looked at burst in acute electrophysiologic preparations in order to explore possible mechanisms of burst waveforms [27,28,29]. We believe our work here should stand among those studies.

Animal model: we used naïve rodents to study the effects of the various SCS strategies. It is clear that ‘healthy’ patients do not receive SCS, and only those with chronic neuropathic pain are candidates for SCS therapy. There is also a long history of preclinical and clinical work suggesting that neural systems undergo significant functional and morphological changes in chronic pain states. Thus, our use of naïve models may limit the applicability of our observations, as these neural responses might differ in chronic pain animals. We do note that a significant amount of preclinical SCS work, considered seminal to SCS mechanistic understanding, has been performed in naïve rodent models [39,40]. Thus, we feel that our work here can also valuably contribute to the SCS knowledge base.

Neuron model: we focused on the responses of NAI and AE neurons in the superficial DH. It is likely simplistic to assume that the activation of inhibitory neurons may lead directly to reduced pain, as DH circuitry is highly complex [41]. Additionally, activation of excitatory neurons might not lead directly to increased excitability of pain-processing neural circuits. Nonetheless, we feel that our general observations that 10 kHz SCS strongly drives an inhibitory response via ostensibly GABAergic neurons in the superficial DH, a structure well-understood to process primarily pain signals, provide a reasonable underpinning for a pain relief hypothesis of 10 kHz SCS.

## 5. Conclusions

We observed, at stimulation intensities that would ostensibly not activate dorsal columns (i.e., ‘paresthesia-free’), that 10 kHz selectively activates non-adapting/inhibitory neurons at higher rates and with more selectivity than passive and asymmetric burst SCS strategies. This suggests that 10 kHz SCS and burst strategies likely operate via different mechanisms.

## Figures and Tables

**Table 1:** Total dorsal horn neuron ‘responders’ for each SCS strategy.

Stimulation Strategy	Non-Adapting/Inhibitory (NAI) *n* = 21	Adapting/Excitatory (AE) *n* = 22
10 kHz	20	2
Passive Burst (PB)	13	11
Asymmetric Burst (AB)	13	19

## Data Availability

The data, analytic methods, and study materials for this trial may be made available to other researchers in accordance with the policies of Nevro Corp.
